# Predicting venous thromboembolism among hospitalized adults: a protocol for development and validation of an implementable real-time prognostic model

**DOI:** 10.1186/s41512-025-00205-8

**Published:** 2025-09-08

**Authors:** Henry J. Domenico, Benjamin F. Tillman, Shari L. Just, Yeji Ko, Amanda S. Mixon, Asli Weitkamp, Jonathan S. Schildcrout, Colin Walsh, Thomas Ortel, Benjamin French

**Affiliations:** 1 Department of Biostatistics, Vanderbilt University Medical Center, Nashville, TN USA; 2 Division of Hematology and Oncology, Department of Medicine, Vanderbilt University Medical Center, Nashville, TN USA; 3 Knowledge Engineering, Health IT, Vanderbilt University Medical Center, Nashville, TN USA; 4 Geriatric Research, Education and Clinical Center, VA Tennessee Valley Healthcare System, Nashville, TN USA; 5 Division of General Internal Medicine and Public Health, Department of Medicine, Vanderbilt University Medical Center, Nashville, TN USA; 6 Department of Medicine, Duke University School of Medicine, Durham, NC USA; 7 Department of Biomedical Informatics, Vanderbilt University Medical Center, Nashville, United States; 8 Department of Medicine, Vanderbilt University Medical Center, Nashville, United States; 9 Department of Psychiatry, Vanderbilt University Medical Center, Nashville, United States

**Keywords:** Clinical prediction model, Model development, External validation, Venous thromboembolism, Electronic health record

## Abstract

**Background:**

Hospital-acquired venous thromboembolism (HA-VTE) is a leading cause of morbidity and mortality among hospitalized adults. Numerous prognostic models have been developed to identify those patients with elevated risk of HA-VTE. None, however, has met the necessary criteria to guide clinical decision-making. This study outlines a protocol for refining and validating a general-purpose prognostic model for HA-VTE, designed for real-time automation within the electronic health record (EHR) system.

**Methods:**

A retrospective cohort of 132,561 inpatient encounters (89,586 individual patients) at a large academic medical center will be collected, along with clinical and demographic data available as part of routine care. Data for temporal, geographic, and domain external validation cohorts will also be collected. Logistic regression will be used to predict occurrence of HA-VTE during an inpatient encounter. Variables considered for model inclusion will be based on prior demonstrated association with HA-VTE and their availability in both retrospective EHR data and routine clinical care. Least absolute shrinkage and selection operator (LASSO) with tenfold cross-validation will be used for initial variable selection. Variables selected by the LASSO procedure, along with those deemed necessary by clinicians, will be used in an unpenalized multivariable logistic regression model. Discrimination and calibration will be reported for the derivation and validation cohorts. Discrimination will be measured using Harrell’s *C* statistic. Calibration will be measured using calibration intercept, calibration slope, Brier score, integrated calibration index, and visual examination of non-linear calibration curve. Model reporting will adhere to the Transparent Reporting of a multivariable prediction model for Individual Prognosis Or Diagnosis guidelines for clinical prediction models using machine learning methods (TRIPOD + AI).

**Discussion:**

We describe methods for developing, evaluating, and validating a prognostic model for HA-VTE using routinely collected EHR data. By combining best practices in statistical development and validation, knowledge engineering, and clinical domain knowledge, the resulting model should be well suited for real-time clinical implementation. Although this protocol describes our development of a model for HA-VTE, the general approach can be applied to other clinical outcomes.

**Supplementary Information:**

The online version contains supplementary material available at 10.1186/s41512-025-00205-8.

## Background

Venous thromboembolism (VTE) most frequently occurs among currently or recently hospitalized individuals [1]. Risk is known to vary by age and sex [1]. Despite increased awareness and efforts at prevention, VTE remains a leading cause of morbidity and mortality among hospitalized adults [2]. In the past 30 years, there have been no significant improvements in rates of VTE, and epidemiological studies suggest the overall incidence is increasing among an older population with a higher burden of comorbidities [3]. Currently available tools for predicting and preventing hospital-acquired VTE (HA-VTE) are limited in application to specific patient subpopulations (e.g., patients requiring medical care not in the intensive care unit, or patients undergoing scheduled surgery), provide overly broad categories for predicted risk based on dichotomized values for continuous risk factors (e.g., age, body mass index), and are difficult to automate in electronic health record (EHR) systems for use in real time [4–8]. Additionally, most of these models unfortunately have exhibited only modest prediction accuracy in derivation and validation cohorts, resulting in insufficient evidence to recommend the use of any specific model over others to guide clinical decision-making [9].

We previously developed and evaluated an initial prognostic model for HA-VTE in a derivation cohort of 132,365 patient encounters at the Vanderbilt University Medical Center (VUMC) adult hospital (1/1/2018–12/31/2020) [10]. While the development of this initial model was limited to demographic and clinical characteristics, diagnostic procedures, vital signs, and laboratory measurements, we demonstrated that such a model could perform well among the entire inpatient population, in contrast with other models that have been developed for specific patient populations. In this study, we will refine the model based on ongoing input from clinicians and consider additional risk factors for HA-VTE (e.g., medications, social determinants of health, environmental factors), with an eye toward real-time implementation. We will also perform additional external validation of the model to assess model generalizability.

Here, we outline the protocol for refining and validating a general-purpose prognostic model for HA-VTE designed for automated implementation within the EHR system. The target population includes all general adult patients admitted to the hospital. The model’s intended purpose is to calculate the predicted probability of HA-VTE for individual patients early in the hospital stay, and to guide HA-VTE prevention strategies. The scores will be made available to healthcare professionals. External validation of the model will be conducted across three distinct domains: temporal validation (in the same population in which the model was developed, but from a different calendar time), geographic validation (in a population different from the derivation population, but with similar characteristics), and domain validation (in a population with characteristics different from those in the derivation population). Model reporting will adhere to the Transparent Reporting of a multivariable prediction model for Individual Prognosis Or Diagnosis using machine learning methods (TRIPOD + AI) guidelines [11].

## Methods

### Setting

Model development and refinement will be performed using a derivation cohort of patients admitted to VUMC, a large academic medical center located in Nashville, TN. The derivation cohort consists of 132,561 inpatient encounters collected between 1/1/2018 and 12/31/2020. Temporal external validation will be conducted using a cohort of patients admitted to VUMC between 1/1/2021 and 10/21/2024. Geographic external validation will involve a cohort of patients admitted to Duke University Medical Center—a large academic medical center that serves a similar patient population as VUMC but in a different geographical location. Domain external validation will use a cohort of patients admitted to Vanderbilt regional hospitals that serve a distinct patient population. This study was approved by the Vanderbilt University Medical Center Institutional Review Board.

### Population

We will include all adult inpatient encounters occurring during the specified locations and timeframes. All patients admitted to the adult inpatient setting over the age of 16 years will be included. Exclusion criteria are kept purposely minimal to maximize generalizability.

### Data sources

Integrating prediction models into clinical workflows requires consistent data definitions between the data used for model development and the data used for real-time implementation. To ensure that the retrospective data extracted for model development aligned with the data available during real-time use, we adopted knowledge engineering best practices. These practices allowed us to accurately represent the clinical concepts to be used by the model and included steps for data coverage analysis and validation.

First, we represented the clinical concepts of interest using standards-based value sets, incorporating applicable standard terminologies such as LOINC, SNOMED, and RxNorm. Each clinical concept to be referenced in the model was defined using these terminologies to ensure standardization. Next, we analyzed the local EHR build to evaluate the coverage of the concepts represented by the standards-based value sets. During this process, any gaps were addressed by augmenting the value sets with local extensions, such as EHR-specific identifiers. Finally, we collaborated with clinical subject-matter experts in a validation step to ensure that the identified concepts accurately captured their intended clinical definitions. Once validated, the value sets were enhanced with appropriate metadata to facilitate ongoing lifecycle management.

These best practices improve consistency between the developed model and its real-time implementation, while enabling semantic interoperability by utilizing standards-based concept representation.

### Potential predictors

A fixed set of candidate predictors for potential inclusion in the prognostic model for HA-VTE has been defined. Variables included as candidate predictors were based on prior demonstrated association with HA-VTE and their availability in both retrospective EHR data and routine clinical care. Candidate predictor categories include demographic characteristics and social determinants of health (e.g., age, sex, race, and ethnicity), inpatient encounter details (e.g., admission type), medical history that is either active or diagnosed during the encounter (e.g., Elixhauser comorbidity details [12], prior history of VTE), vital signs, laboratory measurements, medications (e.g., immunoglobulins, glucocorticoids, statins), nurse-recorded information regarding mobility (e.g., Braden sub-scores, Richmond Agitation-Sedation Scale (RASS) score [13]), and environmental factors. Braden and RASS scores are routinely documented in the nursing flowsheet, with Braden scores typically recorded every shift per hospital policy. A full list of variables and their definitions are provided in the appendix (see Additional file 1). Indicator variables will be created for whether medications within each category were active at admission and administered during the encounter. These variables will be considered separately during the model-development process. Environmental factors include the Neighborhood Atlas’s rankings on socioeconomic disadvantage [14] and air quality, as captured by the Environmental Protection Agency’s air quality index [15]. These community-level factors will be linked to patients based on their admission date and home ZIP code. Distributions of continuous variables will be examined for clinically implausible values. Such values will be treated as missing.

### Outcomes

The primary outcome will be the development of any non-superficial VTE during the course of the inpatient encounter. This outcome will be modeled as a binary variable. The time horizon for prediction begins at hospital admission and continues until discharge. Presence of HA-VTE will be defined using ICD-10 diagnosis codes (see appendix). ICD-based outcome definitions are applied consistently across all patients. In a manual retrospective review of 120 randomly selected HA-VTE cases defined using ICD-based identification, each case was confirmed to be a correct diagnosis [10]. VTE diagnosis codes flagged as present on admission will be excluded from the outcome definition. Clinical subtypes of HA-VTE (e.g., location and severity) will be reported. Subtype-specific predictions will not be obtained. Prior diagnosis of VTE will be used to indicate history of VTE and included as a potential predictor.

### Model fairness

Model performance will be evaluated within patient subgroups defined by demographic characteristics, social vulnerability factors, and specific comorbidities. Variables across which model performance will be compared include patient age, sex, race, ethnicity, and cancer status. In addition to standard measures of discrimination, false-negative rates will also be compared across patient categories [16].

### Missing data

Our primary analysis will use fixed-value imputation for continuous variables, for which missing values are replaced by the median value calculated among patients with observed data. For categorical variables, “missing/unknown” will be included as a separate category. We use these approaches for several reasons. First, due to potentially informative presence (i.e., a laboratory measurement is more likely to be ordered for patients for whom an abnormal value is suspected), data will not be missing at random [17]. Second, while multiple imputation (MI) may provide more accurate imputation in retrospective data [18], when used prospectively, it would likely require additional models to be implemented in real time for prediction of missing values. A sensitivity analysis will be performed comparing performance of models using MI and median-value imputation. A detailed simulation study comparing imputation approaches for real-time prediction will be reported separately.

### Statistical analysis

Patient encounter data will be summarized using median and interquartile range (IQR) for continuous variables and counts with percent for categorical variables. Encounter data will be summarized by cohort (derivation, temporal external validation, geographic external validation, and domain external validation) and by HA-VTE status. The rate of HA-VTE will be examined over continuous time and compared between cohorts. Statistical comparisons will be performed using chi-square tests for categorical variables and Kruskal–Wallis tests for continuous variables. A two-sided *p* value < 0.05 will indicate statistical significance. No adjustments will be made for multiple comparisons.

Model development will be performed within the derivation cohort described above. Data from validation cohorts will be kept fully external. Least absolute shrinkage and selection operator (LASSO) regression with tenfold cross-validation will be used for initial variable selection [19]. This will select the subgroup of variables minimizing cross-validation error. We will use the *C* statistic as the error measure to be optimized in the cross-validation process. LASSO variable selection will be performed using the “glmnet” package in R [20]. Restricted cubic splines will be considered for continuous variables. Variables selected in the LASSO procedure will be used in an unpenalized multivariable logistic regression model. Physician feedback regarding model complexity and variables necessary for inclusion will also be incorporated into the identification of the final prognostic model.

Performance of the unpenalized logistic regression model, fit using variables selected in the cross-validation procedure, as well as any additional variables identified by clinicians as necessary for clinical validity, will be reported in the derivation cohort as well as the temporal, domain, and geographic external validation cohorts. More complex models, such as random forest or XGBoost, will not be considered as there was minimal to no improvement in performance when using these models compared to logistic regression (data not shown). Predicted probabilities for each patient will be generated using the fitted logistic regression model object and the *predict()* function in R. Prediction performance will be quantified using measures of discrimination (*C* statistic [21]), calibration (calibration intercept, calibration slope, Brier score, integrated calibration index, and visual examination of non-parametric calibration curve), and distribution of predicted risk [22–24]. Model variable importance and odds ratios with 95% CI will be reported. Additionally, we will report model performance within pre-specified subgroups. Predictions in the external cohorts will be generated using the non-recalibrated model. Models with recalibrated coefficients may be considered if there is a substantial decrement in prediction performance. In such cases, recalibrated and non-recalibrated model performance will be reported and compared. We will follow TRIPOD + AI guidelines [11] for model reporting. All analyses will be conducted using the current version of R statistical software [25].

## Discussion

This protocol describes the rationale and methodology for refining and validating a prognostic model for HA-VTE suitable for real-time clinical implementation. We describe a strategy for temporal, geographic, and domain external validation. Additionally, we describe a strategy for comparing model performance across patient subgroups and evaluating consistency in model performance across subgroups. Our approach is generalizable to the development of clinical prediction models for other outcomes.

### Strengths and limitations

Strengths of our approach include large cohorts representative of a general inpatient population for both model development and validation. Our cohorts also include a wide array of clinical variables that are routinely collected and available for real-time prediction of HA-VTE. Working directly with biomedical informatics experts, we have ensured that data definitions between historical and real-time data elements will be consistent. This consistency will ensure that the prognostic models can be directly translated into clinical workflows and should lead to improved prospective model performance.

Our approach has several limitations that should be acknowledged. First, the derivation cohort only includes patients admitted to a single large academic medical center, potentially limiting generalizability. Second, the primary outcome is limited to development of HA-VTE during the time of admission to the hospital and does not capture events occurring post-discharge. Third, we rely on ICD-10 code definitions for our outcome definition, which could lead to misclassification or underestimation of the overall incidence. Fourth, fixed-value imputation will be used in cases where data is missing or incomplete. More complex methods for imputation, such as multiple imputation, could be used. However, use of these methods may limit the prospective clinical utility of the derived model. Additionally, inpatient interventions—including initiation of thromboprophylaxis—may reduce the incidence of HA-VTE in high-risk patients, which can influence model calibration and discrimination. To avoid introducing confounding by indication, we chose not to include prophylactic interventions as candidate predictors. Finally, we acknowledge that, on its own, providing the predicted probability of HA-VTE for each patient will not improve outcomes. The developed model will be deployed in real time and evaluated as part of a pragmatic randomized controlled trial aimed at reducing HA-VTE among hospitalized adults.

## Conclusion

In this protocol, we outline a strategy for refining and validating a clinical prediction model combining rigorous statistical development and validation methods, knowledge engineering best practices, and clinical domain knowledge. The resulting model should be well suited for real-time clinical implementation. Although this protocol describes our development of a model for HA-VTE, the general approach can be applied to other clinical outcomes.

## Supplementary Information

Supplementary Material 1.

## Data Availability

No datasets were generated or analysed during the current study.

## Abbreviations

ADI: Area deprivation index
AI: Artificial intelligence
AQI: Air quality index
AUC: Area under receiver operating curve
EHR: Electronic health record
HA-VTE: Hospital-acquired venous thromboembolism
ICD: International classification of diseases
IQR: Interquartile range
LASSO: Least absolute shrinkage and selection operator
LOINC: Logical observation identifiers, names, and codes
MI: Multiple imputation
RASS: Richmond Agitation-Sedation Scale
SNOMED: Systematized nomenclature of medicine
TRIPOD: Transparent Reporting of a multivariable prediction model for Individual Prognosis Or Diagnosis
VUMC: Vanderbilt University Medical Center
